# Epitope Mapping of BmpA and BBK32 *Borrelia burgdorferi* Sensu Stricto Antigens for the Design of Chimeric Proteins with Potential Diagnostic Value

**DOI:** 10.1021/acsinfecdis.3c00258

**Published:** 2023-10-07

**Authors:** Weronika Grąźlewska, Lucyna Holec-Gąsior, Karolina Sołowińska, Tomasz Chmielewski, Beata Fiecek, Marinela Contreras

**Affiliations:** † Department of Molecular Biotechnology and Microbiology, Faculty of Chemistry, University of Gdańsk Technology, 80-233 Gdańsk, Poland; ‡ SaBio, Instituto de Investigación en Recursos Cinegéticos IREC−CSIC-UCLM-JCCM, 13005 Ciudad Real, Spain; § Department of Parasitology and Diseases Transmitted by Vectors, National Institute of Public Health NIH - National Research Institute, 00-791 Warsaw, Poland

**Keywords:** Lyme disease, Borrelia burgdorferi sensu lato, epitope mapping, serodiagnosis, chimeric proteins

## Abstract

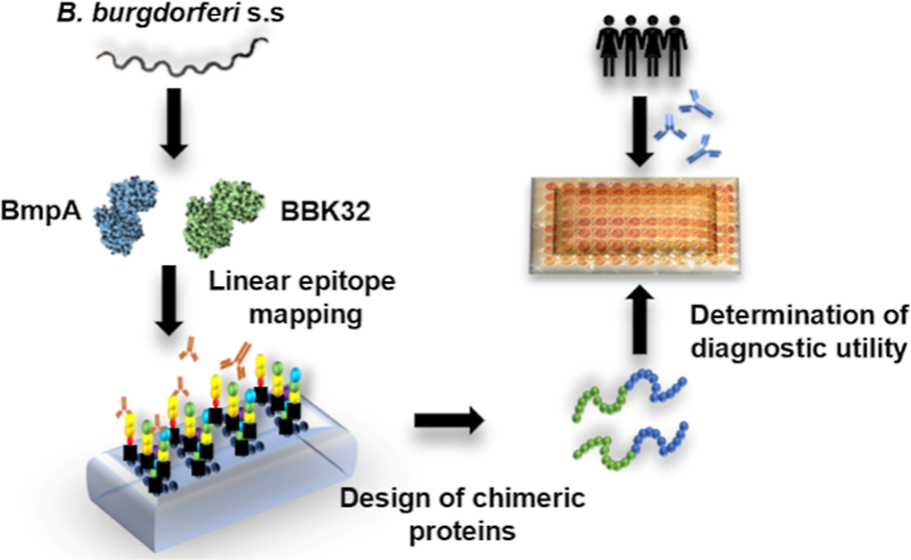

Lyme disease is a tick-borne zoonosis caused by Gram-negative bacteria belonging to the *Borrelia burgdorferi* sensu lato (s.l.) group. In this study, IgM- and IgG-specific linear epitopes of two *B. burgdorferi* sensu stricto (s.s.) antigens BmpA and BBK32 were mapped using a polypeptide array. Subsequently, two chimeric proteins BmpA-BBK32-M and BmpA-BBK32-G were designed to validate the construction of chimeras using the identified epitopes for the detection of IgM and IgG, respectively, by ELISA. IgG-ELISA based on the BmpA-BBK32-G antigen showed 71% sensitivity and 95% specificity, whereas a slightly lower diagnostic utility was obtained for IgM-ELISA based on BmpA-BBK32-M, where the sensitivity was also 71% but the specificity decreased to 89%. The reactivity of chimeric proteins with nondedicated antibodies was much lower. These results suggest that the identified epitopes may be useful in the design of new forms of antigens to increase the effectiveness of Lyme disease serodiagnosis. It has also been proven that appropriate selection of epitopes enables the construction of chimeric proteins exhibiting reactivity with a specific antibody isotype.

Lyme disease is a multisystem disorder most often affecting the nervous system, cardiac system, joints, and skin. The causative agent is spirochetes belonging to the *Borrelia burgdorferi* sensu lato (s.l.) complex, transmitted to humans by *Ixodes* ticks. So far, pathogenicity for humans in Europe has been unquestionably confirmed for 5 genospecies, i.e., *Borrelia afzelii*, *Borrelia bavariensis*, *B. burgdorferi* sensu stricto (s.s.), *Borrelia garinii,* and *Borrelia spielmanii*. In North America, almost all cases are caused by *B. burgdorferi* s.s.; however, there are some reports of *Borrelia mayonii* infections.^1,2^

The only characteristic symptom of Lyme disease is erythema migrans (EM), which does not appear in all infected people and sometimes takes a nonspecific form. For these reasons, the diagnosis of Lyme disease is based on laboratory techniques, mainly by serodiagnosis. At present, a two-tier serological test is recommended in most countries. First, an enzyme-linked immunosorbent assay (ELISA) is carried out, and in the event of a positive or inconclusive result, Western blot (WB) is performed as a confirmatory test. In WB, only the reactivity of specific antibodies with antigens strictly defined for immunoglobulins M and G is taken into account.^3−5^

Despite the fact that the standard, especially in the later stages of infection, is serodiagnosis, it has some limitations, mainly caused by the complicated antigenic structure of *B. burgdorferi* s.l. The use of whole-cell lysates (WCL) or antigens of one genospecies may not be sufficient for the proper diagnosis of Lyme disease due to the large number of genospecies within the *B. burgdorferi* s.l. group as well as the relatively low degree of amino acid sequence conservation in their proteins. This problem is particularly relevant in Europe, where many *B. burgdorferi* s.l. genospecies pathogenic to humans occur.^6^ What is more, a large diversity of antigenic structures between isolates as well as different stages of spirochete growth are observed. Some plasmids are lost during in vitro cultivation and with them, the antigens coded by them. This makes it very difficult to obtain cell lysates with a standardized composition and affects the repeatability of enzyme immunoassay results.^7,8^

Moreover, *B. burgdorferi* s.l. contains many proteins that are homologues of antigens present in other pathogens, which may lead to cross-reactions. The frequent nonspecific reactions were the reason for the introduction of the two-step serodiagnostic test, as ELISA alone was not sufficiently specific. The introduction of a second-stage WB test improved the specificity of diagnostics but also significantly increased its costs.^5,9,10^

The use of recombinant proteins is a potential solution to the problems of Lyme disease serodiagnosis. At present, they are the main form of antigens used in commercial WB (EUROLINE Borrelia-RN-AT (Euroimmun), recomLine Borrelia IgG/IgM (Microgen)). In ELISA, it is common to add one or more recombinant proteins to the WCL (Anti-Borrelia plus VlsE (Euroimmun)); however, commercial ELISA based solely on recombinant proteins or synthetic peptides are also available, for instance, ZEUS ELISA Borrelia VlsE1/pepC10 IgG/IgM Test System (ZEUS Scientific), C6 Lyme ELISA kit (Immunetics), and Anti-Borrelia Select ELISA (Euroimmun).^1^ Due to *B. burgdorferi* s.l. being characterized by slow growth and its requirement for an expensive growth medium, producing recombinant proteins using an expression system based on *Escherichia coli* is a more cost-effective and simpler method for obtaining antigens compared to whole-cell lysates production.^1,11,12^ In addition, careful and thoughtful antigen selection can reduce cross-reactivity and allow test sensitivity to be independent of the *B. burgdorferi* s.l. genospecies that caused the infection, which will simplify the interpretation of diagnostic assays. For this purpose, antigens or their fragments that are conserved within the *B. burgdorferi* s.l. group, as well as those that cause cross-reactivity, should be identified.

Enzyme immunoassays based on single recombinant proteins may have low sensitivity, as there are hundreds of antigens in the WCL that can be recognized by specific antibodies. By using single proteins, the number of epitopes that interact with immunoglobulins is significantly reduced. Higher immunoassay sensitivity can be achieved through the use of chimeric proteins that contain selected immunodominant fragments from several proteins in a single amino acid chain. This means that such a protein could be recognized by antibodies specific to several antigens.^13,14^

B-cell epitope mapping appears to be a key step in the rational design of chimeric proteins. It enables the identification of highly specific epitopes and sequences responsible for cross-reactions, which allows the selection of appropriate fragments for the construction of multivalent proteins. There are several methods for mapping conformational and linear epitopes where advances in genomics, proteomics, and computational methods have contributed significantly to the development of immunoinformatics. Computational methods allow identification of epitopes and prediction of the antibody structure. Despite the fact that an in silico approach saves time and money, it is advisable to confirm the obtained results by experimental methods.^15^ X-ray crystallography of antigen–antibody complexes is believed to be the most accurate method for mapping linear and structural epitopes, but regardless of its undeniable advantages, this technique is not used routinely because it is quite complicated and expensive.^16^

Peptide microarrays are the most popular method for mapping linear B-cell epitopes. This approach allows for the determination of sequences recognized by specific antibodies in a complete antigen sequence. Peptide microarrays are composed of many short, overlapping peptides (15–20 amino acids) printed on a solid surface. This is a quick and low-cost method, as it allows the analysis of thousands of peptides simultaneously. Therefore, it is currently the basis of many studies aimed at identifying new proteins with diagnostic or immunoprotective utility.^17^

This study aimed to determine whether epitope mapping can contribute to improving the serodiagnosis of Lyme disease by designing chimeric proteins from BmpA and BBK32 proteins. For this purpose, linear epitopes of BmpA and BBK32 *B. burgdorferi* s.s. B31 antigens were mapped by using peptide microarrays. These proteins were selected because both are surface lipoproteins responsible for adhesion to host cells, and their production begins when the tick takes a meal from the blood, so these antigens should be well exposed to the immune system.^18,19^ In addition, the literature reports their reactivity with anti-*B. burgdorferi* s.l. antibodies,^20−23^ and as a result, immunodominant epitopes were found. To determine whether knowledge of the distribution of epitopes in the antigen sequence can contribute to the improvement of serodiagnostics, two chimeric proteins containing highly specific immunodominant fragments were designed, one of them intended for the detection of IgG and the other IgM.

## Results

### Reactivity of Serum Samples against BBK32 and BmpA Peptides

**Figure 1:**
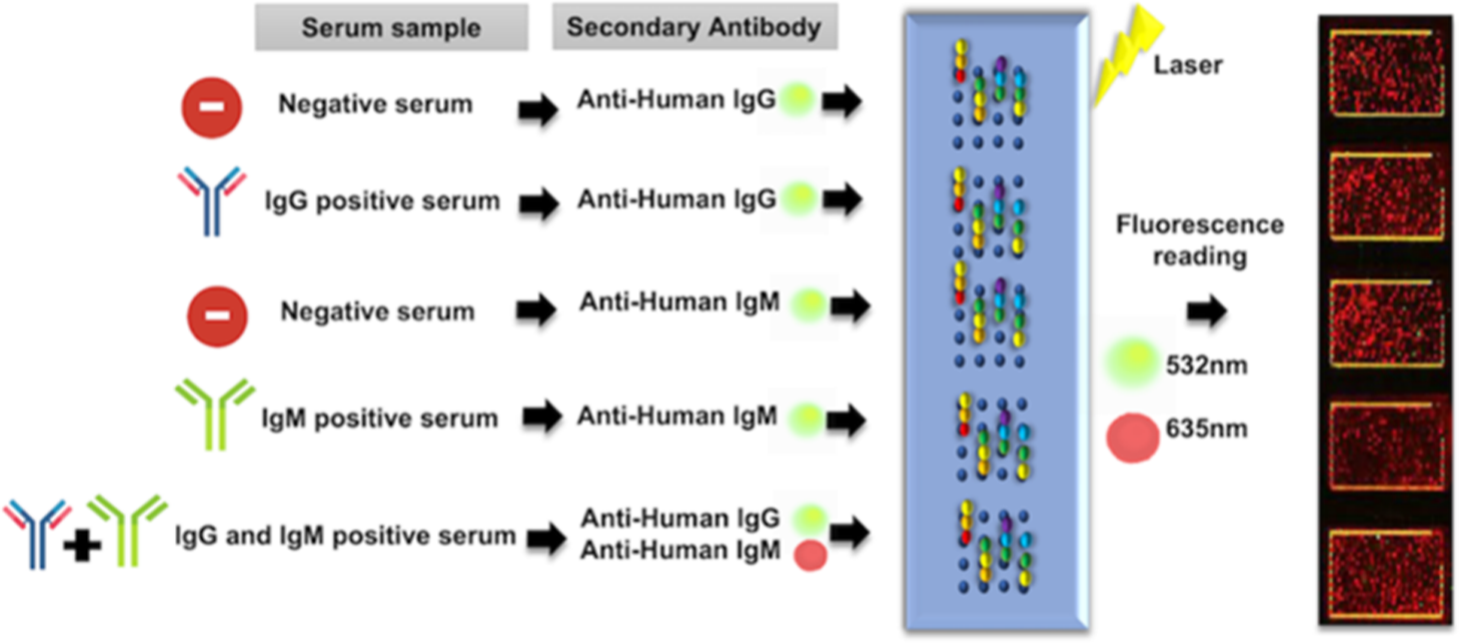
Experimental design of the study. The samples used in peptide mapping were from human serum samples positive for IgG, positive for IgM, positive for both immunoglobulins, and negative. A peptide microarray was performed for the identification of BmpA and BBK32 reactive epitopes or reactive peptide regions recognized by IgG and IgM antibodies from the serum groups.

Scan peptide microarrays containing aa sequences from *B. burgdorferi* s.s. B31 proteins BBK32 and BmpA were incubated with serum samples positive for either IgG antibodies or IgM antibodies, as well as sera positive for both immunoglobulin groups and negative controls (Figure 1).

**Figure 2:**
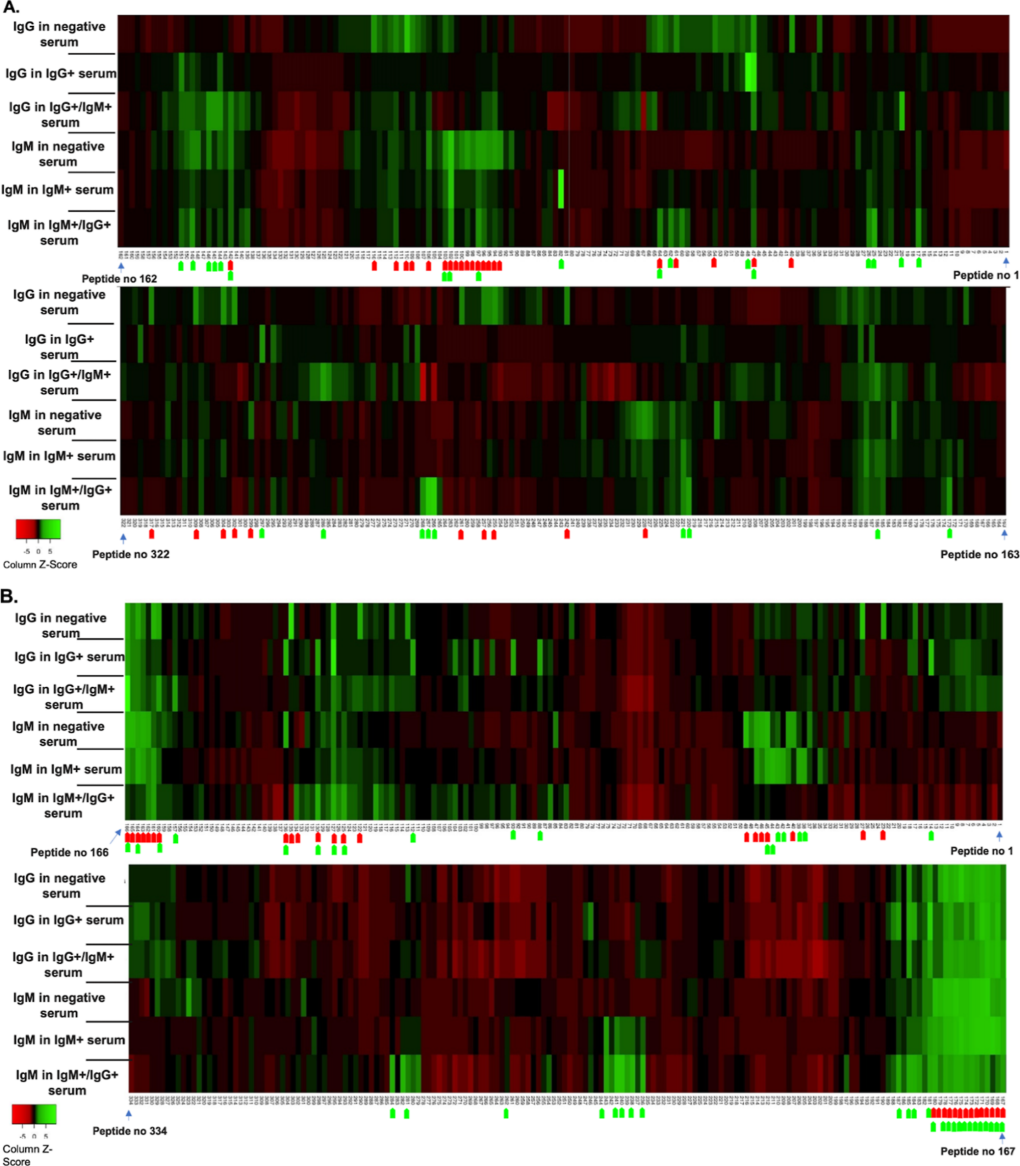
Heatmap of the IgG and IgM reactive epitopes in positive IgG, positive IgM, positive IgG/IgM, and negative serum samples in (A) BmpA and (B) BBK32 proteins. Reactivity against peptides is indicated with a *Z*-score, and possible epitope regions are identified with a green arrow when *Z*-score > 2. Red arrows show peptides with significant reactivity with pooled sera from the control groups.

Reactive epitopes with a *Z*-score > 2 are highlighted with arrows on the bottom of each heatmap (Figure 2). A bright green arrow corresponds to regions of highly reactive peptides recognized by IgG or IgM in positive serum samples, whereas a bright red arrow points to peptides recognized by immunoglobulins from negative serum samples. Some regions were reactive for immunoglobulins from both negative and positive serum samples, which indicates cross-reactive peptide regions. For example, in BBK32 an extensive region of the protein (overlapping peptides from number 160 to 180) is recognized by IgG and IgM from both positive and negative serum samples. Other isolated peptides were also reactive to both positive and negative sera; however, a *Z*-ratio > 1.96 was only found for positive samples. In BmpA, this pattern of reaction is less frequent, although some peptides, such as 47, are recognized by IgG in both positive and negative sera with a *Z*-score > 2, yet this peptide is also significantly more intensely recognized in samples from positive patients with a *Z*-ratio > 1.96. Peptides 97, 102, and 103 are also recognized by IgM in positive and negative sera (Figure 2a, Table S1 in the Supporting Information). Peptides significantly reactive for a single immunoglobulin isotype were observed in positive serum samples, such as peptide 20 in BmpA, KVSLIIDGTFDDKSF, which was reactive to IgG in the mixed (IgG+/IgM+) positive samples or peptide 26, DGTFDDKSFNESALN, which was only recognized by IgM from positive samples (Table S1). In the same protein, a peptide region including the overlapping peptides 265–268 is reactive and recognized by IgM from IgG+/IgM+ samples (Table S1, Figure 2a).

In BBK32, a peptide region with overlapping peptides, from 38 to 45, was reactive only to IgM with a *Z*-ratio > 1.96 when compared with the control group (IgM in negative samples). In addition, in this same protein the peptides 14, 88, 93, 112, 130, and 136 were reactive (*Z* score > 2) and recognized only by IgG in the positive serum samples and showed a *Z*-ratio > 1.96 when compared with control (IgG in negative samples) (Table S1, Figure 2b).

In IgG+/IgM+ serum samples, some peptides could significantly differentiate positive samples from negative samples for only one immunoglobulin type. This is observed in BmpA, as mentioned above, in peptide 20 (Figure 2a). Peptide-overlapping regions of peptides 142–146, 186, and 286 were recognized by IgG, and peptides 25, 62, 64,149, 173, and 220 were recognized by IgM (Table S1). In protein BBK32, peptides such as 125, 127, 130, 185, 282, and 284 were recognized by IgM in IgG+/IgM+ serum samples, while peptides recognized by IgG in IgG+/IgM+ serum samples, allowing for significant differentiation between IgG positive and IgG negative samples, were not found (Table S1).

### Design of Chimeric Proteins for IgG and IgM Detection

As a proof of concept that epitope mapping of these two proteins is a tool to improve diagnosis in patients exposed to *B. burgdorferi* s.l., two chimeric proteins, one for IgM (BmpA-BBK32-M) and one for IgG (BmpA-BBK32-G) detection in serum samples, were designed using peptides or regions containing significant reactive epitopes (*Z*-ratio > 1.96) when compared to the epitopes recognized by the control samples that were conserved in the *B. burgdorferi* s.l. complex and that excluded cross-reactive regions.

**Figure 3:**
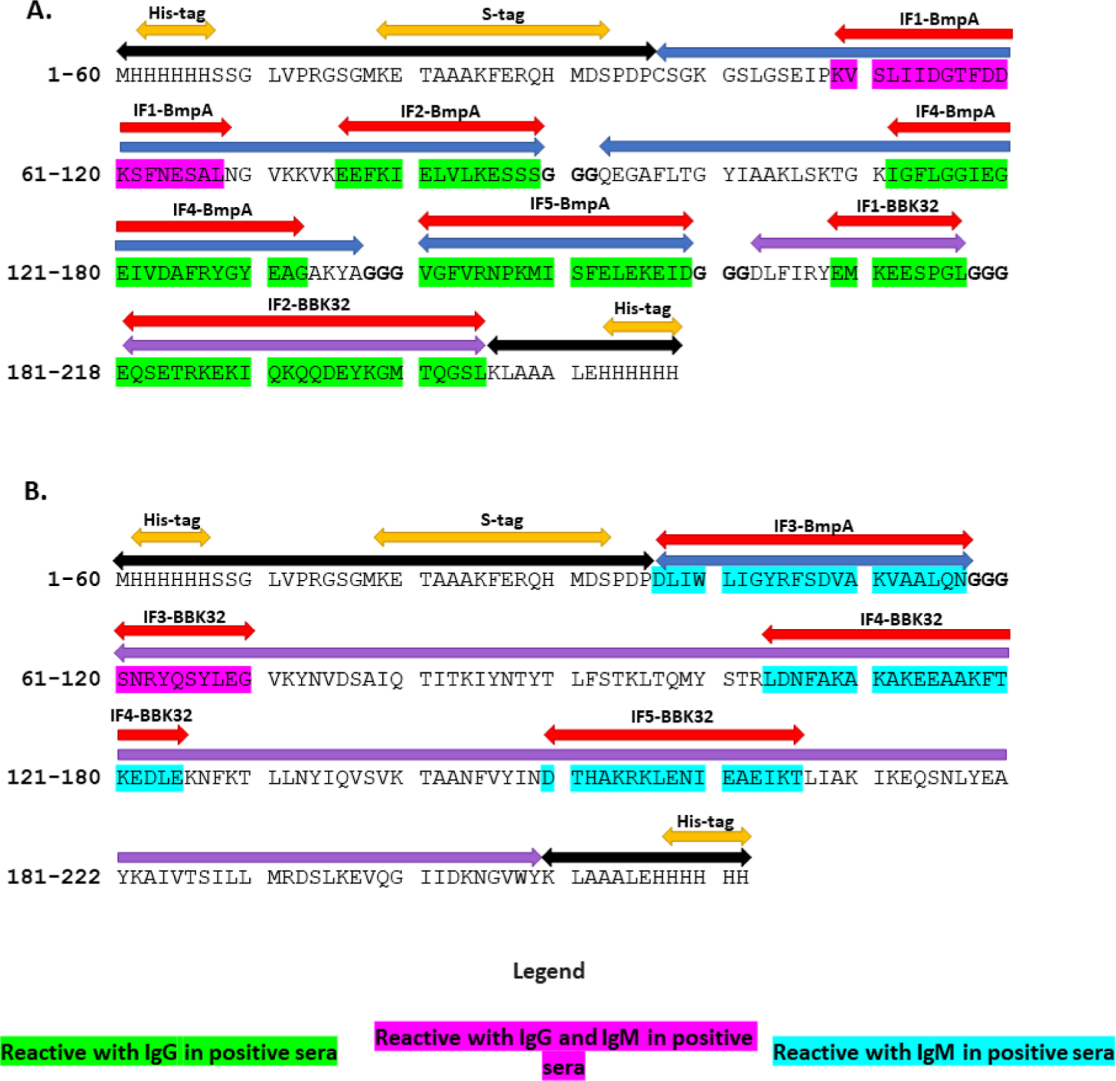
Complete amino acid sequence of designed (A) BmpA-BBK32-G and (B) BmpA-BBK32-M. Red arrows indicate immunodominant fragments, blue arrows sequences derived from BmpA, purple arrows sequences derived from BBK32; black arrows sequences derived from the pUET1 vector, orange arrows specific domains coding by pUET1 vector, and -GGG- separating individual fragments are in bold. IF = immunodominant fragments containing the specific identified epitopes.

For this purpose, peptides significantly reactive (*Z*-ratio > 1.96) for IgG immunoglobulin isotype, in positive IgG and IgG+/IgM+ serum samples, were selected. The peptides included in the BmpA-BBK32-G chimeric protein were 20, 47, 144–151, and 286 from BmpA and 14, 93, 100, and 112 from BBK32 (Tables S1 and S2 in the Supporting Information). To increase antigen specificity, peptides with large overlapping regions that include peptides recognized by IgM or immunoglobulins from negative serum samples were eliminated or not selected for the design. The constructed chimeric protein BmpA-BBK32-M, for IgM detection, included reactive peptides recognized in positive IgM and IgG+/IgM+ serum samples including peptides 64 and 82 from BmpA and 185, 236–241, 243, 282, and 284 from BBK32 (Tables S1 and S2). Furthermore, nonreactive fragments were also introduced into the chimeric protein sequences to ensure the proper molecular mass for their efficient biotechnological production. In addition, the sequence -GGG- amino acids spacer was introduced between individual fragments to better expose these epitopes (Figure 3).

### Analysis of the Amino Acid Sequence of Identified Fragments

The amino acid sequence identity of BmpA-specific immunodominant fragments (IF-BmpA) in the complex *B. burgdorferi* s.l. was high, ranging from 100 to 79% (Table 1). As the sequence used for epitope mapping was derived from *B. burgdorferi* s.s. B31, it was in this genospecies that the highest degree of sequence identity was observed. The best-conserved immunodominant fragment was IF-4-BmpA, and its identity was at least 95% in all genospecies. The IF-2-BmpA and IF-3-BmpA peptides had the lowest sequence identity but always around 80%.

**Table 1:** Degree of Conservation of Immunodominant Fragments Identified in BmpA within *B. burgdorferi* s.l

				sequence identity				
IF no.	sequence of immunodominant fragments	reactive Ab isotype	length [aa]	*Bb* s.s.^a^ (%)	*Ba*^b^ (%)	*Bg*^c^ (%)	*Bbv*^d^ (%)	*Bs*^e^ (%)
**1**	^30^KVSLIIDGTFDDKSFNESAL^49^	IgM/IgG	20	100	95	85	85	100
**2**	^57^EEFKIELVLKESSS^70^	IgG	14	100	86	93	93	79
**3**	^86^DLIWLIGYRFSDVAKVA^102^	IgM	17	94–100	88	82	82	100
**4**	^154^IGFLGGIEGEIVDAFRYGYEAG^175^	IgG	22	100	95	95–100	100	95
**5**	^293^VGFVRNPKMISFELEKEID^311^	IgG	19	100	89	89	89	95

a *B. burgdorferi* s.s. (strains B31; JD1; 156a; N40).

b *B. afzelii* (strains: PKo; K78; ACA-1; A91).

c *B. garinii* (strains: 20047; 50; BgVir; 40).

d *B. bavariensis* (strain: Pbi).

e *B. spielmanii* (strain: A14S).

The amino acid sequence identity of BBK32-derived peptides in the complex *B. burgdorferi* s.l. was slightly lower, ranging from 100 to 67% (Table 2). Sequence differences between individual strains within genospecies were also more often observed. Again, the highest degree of sequence identity was observed for *B. burgdorferi* s.s. It was clearly noticeable that the genospecies in which the selected immunodominant fragments were the most diverse was *B. spielmanii*. The degree of sequence identity for IF-2-BBK32 and IF-4-BBK32 was 67%. However, for the rest of the genospecies, the degree of sequence identity was much higher and fell below 80% for only some strains of *B. afzelii*.

**Table 2:** Degree of Conservation of Immunodominant Fragments Identified in BBK32 within *B. burgdorferi* s.l

				sequence identity				
IF no.	sequence of immunodominant fragments	reactive Ab isotype	length [aa]	*Bb* s.s.^a^ (%)	*Ba*^b^ (%)	*Bg*^c^ (%)	*Bbv*^d^ (%)	*Bs*^e^ (%)
**1**	^27^EMKEESPGL^35^	IgG	9	100	75–89	89–100	89	67
**2**	^110^EQSETRKEKIQKQQDEYKGMTQGSL^134^	IgG	25	96–100	80–88	96–100	84	76
**3**	^2065^SNRYQSY^212^	IgG/IgM	7	100	86	100	100	86
**4**	^249^LDNFAKAKAKEEAAKFTKEDLE^270^	IgM	22	95–100	82–86	91–100	86	100
**5**	^295^DTHAKRKLENIEAEIKT^311^	IgM	17	94–100	78–89	82	82	67

a *B. burgdorferi* s.s. (strains B31; JD1; 156a; N40).

b *B. afzelii* (strains: PKo; K78; ACA-1; A91).

c *B. garinii* (strains: 20047; 50; BgVir; 40).

d *B. bavariensis* (strain: Pbi).

e *B. spielmanii* (strain: A14S).

Sequences that overlap by at least 6 amino acids with those identified as cross-reacting have been also found in many human pathogens, not only prokaryotes (Tables S3 and S4 in the Supporting Information). In this additional analysis, the group of bacteria showing sequence similarity with almost all identified fragments recognized nonspecifically by antibodies were Relapsing Fever *Borrelia* (RFB) bacteria. These sequences also often coincided with fragments of *E. coli* proteins. In this analysis, cross-reactive fragment 5 from BBK32 (CRF-5-BBK32) showed sequence similarity to the greatest number of pathogens (Table S4). CRF-5-BBK32 contains overlapping peptide numbers 160 to 180 and is perfectly visible on the heatmap as an extensive fragment showing high reactivity with all groups of sera (Figure 2b.)

### Expression and Purification of Chimeric Proteins

**Figure 4:**
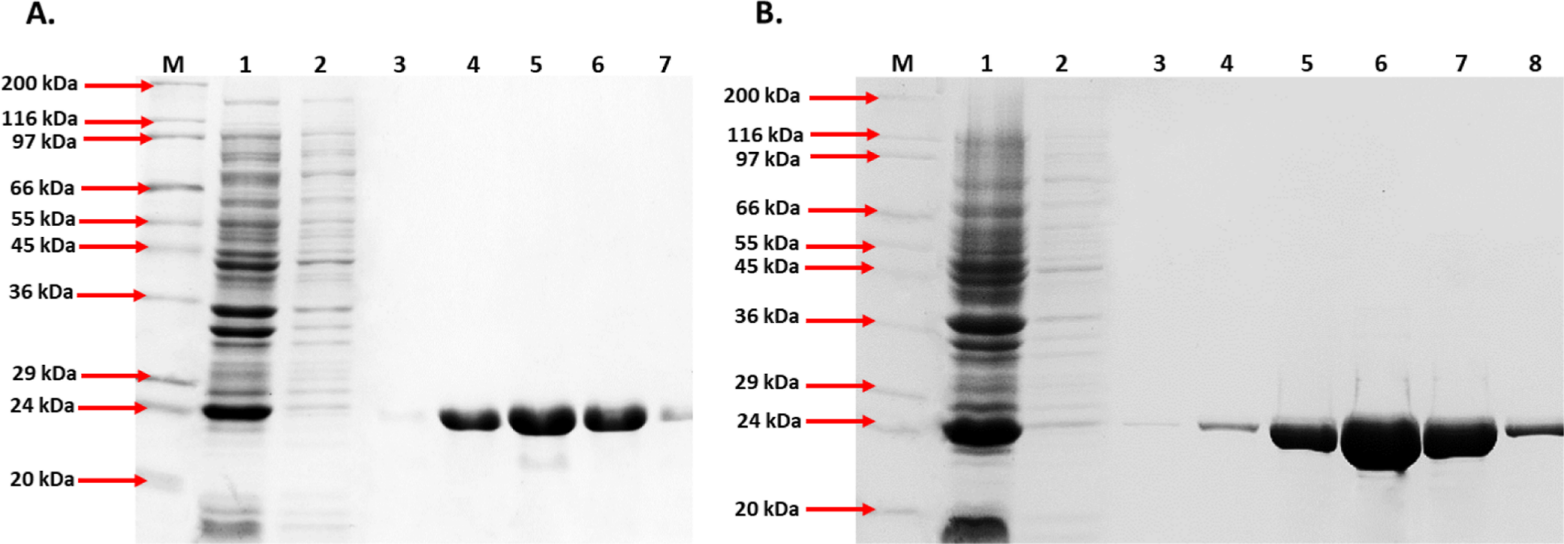
Results of purification of chimeric proteins (A) BmpA-BBK32-G and (B) BmpA-BBK32-M. Lanes (1) *E. coli* BL21(DE3) pLysS lysate before loading into the affinity column; (2) *E. coli* BL21(DE3) pLysS lysate after passing through the affinity column; (3) First elution fraction; (4) Second elution fraction; (5) Third elution fraction; (6) Fourth elution fraction, (7) Fifth elution fraction, and (8) Sixth elution fraction.

B/32-G recombinant antigen was obtained as a chimeric protein with a molecular weight of 24 kDa and an isoelectric point of 6.35. The molecular weight of B/32-M was 25 kDa, and the isoelectric point was 9.2. Electrophoretic purity of the preparations obtained by metal affinity chromatography was above 96% (results not shown). The constructed prokaryotic expression system allowed to obtain approximately 56 and 53 mg of purified B/32-G and B/32-M proteins per liter of culture, respectively. Complete amino acid sequences of the designed chimeric proteins are shown in Figure 3. Results of protein purification are presented in Figure 4 and in the Supporting Information (Figures S1 and S2).

### ELISA

**Figure 5:**
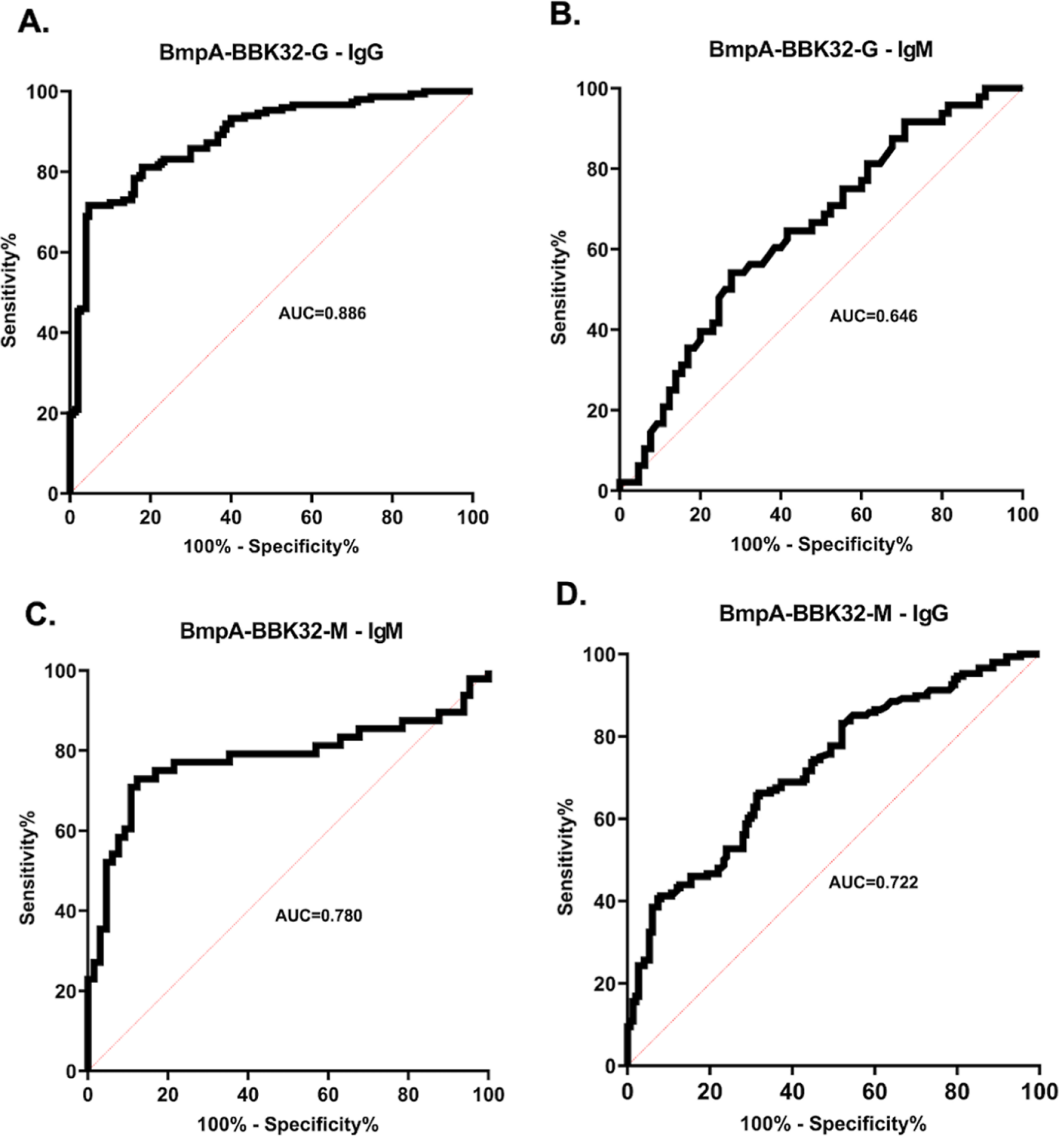
ROC analysis and AUC for (A) IgG-ELISA-B/32-G; (B) IgM-ELISA-B/32-G; (C) IgM-ELISA-B/32-M; and (D) IgG-ELISA-B/32-M.

The sensitivity and specificity of ELISA based on the B/32-G and B/32-M recombinant chimeric proteins were determined using cutoff values obtained by ROC analysis (Figure 5, Table 3). The optimal cutoff values were 0.228, 0.223, and 0.114 for IgG-ELISA-B/32-G, IgM-ELISA-B/32-M, and IgG-ELISA-B/32-M, respectively. The highest sensitivity (71%) and specificity (95%) were shown by IgG-ELISA-B32/G, and what is more, the AUC (0.886) was also the highest. IgM-ELISA-B/32-M showed identical sensitivity (71%); however, its specificity (89%) and especially AUC (0.780) were lower. IgG-ELISA-B/32-M showed noticeably lower sensitivity and specificity.

**Table 3:** Results of IgG and IgM ELISA Based on Chimeric Proteins

protein	detected Ab isotypes	optimal cutoff	sensitivity [ %]	specificity [ %]	AUC	average absorbance	median absorbance
B/32-G	IgG	0.228	71 (105/148)^a^	95 (7/150)^a^	0.886	P^b^: 0.366	P^b^: 0.330
						N^c^: 0.126	N^c^: 0.107
B/32-G	IgM	no statistical difference in absorbance for positive and negative sera (*p* > 0.05)			0.646	P^b^: 0.199	P^b^: 0.177
						N^c^: 0.162	N^c^: 0.141
B/32-M	IgM	0.223	71 (34/48)^a^	89 (7/65)^a^	0.780	P^b^: 0.353	P^b^: 0.240
						N^c^: 0.184	N^c^: 0.170
B/32-M	IgG	0.114	66 (97/148)^a^	69 (47/150)^a^	0.722	P^b^: 0.168	P^b^: 0.136
						N^b^: 0.109	N^b^: 0.098

a Number of seropositive sera/number of tested sera.

b Positive sera.

c Negative sera.

**Figure 6:**
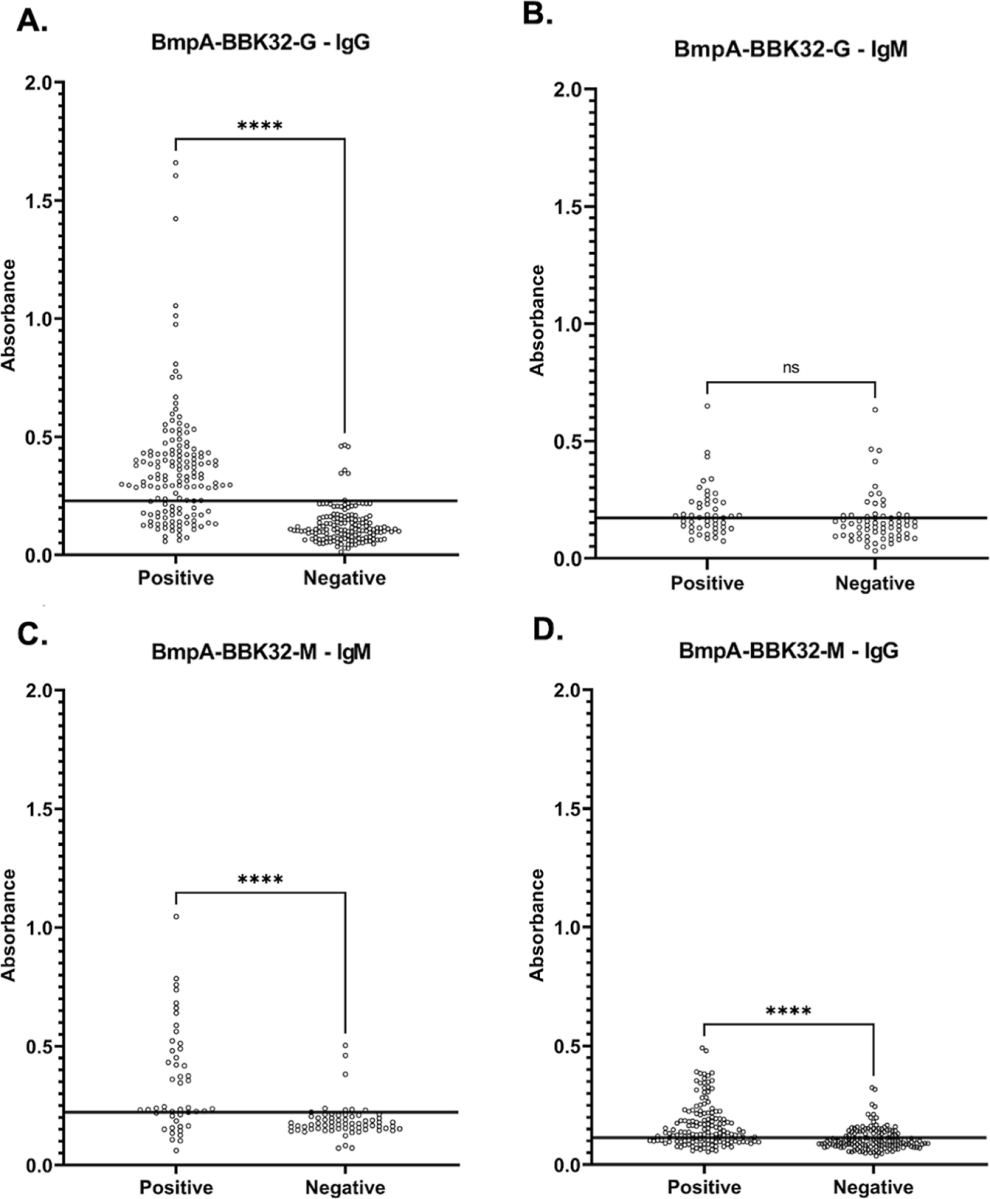
Absorbance values for negative and positive sera for (A) IgG-ELISA-B/32-G; (B) IgM-ELISA-B/32-G; (C) IgM-ELISA-B/32-M; and (D) IgG-ELISA-B/32-M.

Student’s *t*-test showed a statistically significant difference in absorbance between the negative and positive sera for IgG-ELISA-B/32-G, IgM-ELISA-B/32-M, and IgG-ELISA-B/32-M (*p* < 0.0001). For IgM-ELISA-B/32-G, *p* = 0.0634, which proves no statistically significant difference in the absorbance values for the two groups of sera (Figure 6).

## Discussion

Lyme disease affects hundreds of thousands of people in the northern hemisphere each year. The severity of this problem is evidenced by the fact that many countries carry out epidemiological surveillance of Lyme disease.^24^ Despite ongoing efforts, it is not possible to fully control the disease. There is no vaccine available on the market that effectively protects against infection, and there are still many problems associated with accurate diagnosis. Currently, the basis for diagnosis is two-tiered serological testing, but unfortunately, due to the need to perform two serological assays, the cost of this approach is quite high. In addition, interpretation of WB results is subject to the subjective assessment of the diagnostician, which may result in discrepancies between laboratories.^1,25^

Recombinant proteins have been used in commercial assays for many years to enable the diagnosis of infection caused by various genospecies of *B. burgdorferi* s.l.^1,26^ However, the study of new tools or approaches seems necessary to improve Lyme disease serodiagnosis using recombinant proteins, which would increase its specificity and sensitivity while also reducing costs. The hypothesis behind these experiments is to rationally design diagnostically valuable chimeric proteins containing fragments of BmpA and BBK32 antigens based on linear epitope mapping data.

BBK32 and BmpA antigens are extensively used for diagnostics,^21−23^ and in this study, epitope mapping evidenced their value by identifying epitopes or reactive peptide regions with potential diagnostic utility. Previous studies have demonstrated the application of epitope mapping by microarray as a tool that allows the design of chimeric proteins, which improves the recognition and exposure of epitopes to be recognized by IgG antibodies.^27−29^ In addition, B-cell linear epitope mapping could also be useful for the identification of epitopes essential for the development of effective and target-specific vaccines.^28,30^

This study has the added value of identifying possible cross-reactive regions. Ten fragments (5 for both BmpA and BBK2 proteins), containing linear epitopes recognized by antibodies from negative sera, were identified. In fact, the protein that presented the most cross-reactive region was BBK32, where the overlapping peptide region from 160 to 180 was reactive for IgG and IgM both in positive and negative serum samples (Figure 2b, Table S1). All identified cross-reacting fragments contain linear epitopes also effectively recognized by antibodies contained in positive sera (*Z*-score > 2); however, *Z*-ratio did not differ statistically significantly when comparing both groups of sera–negative and positive (*Z*-ratio < 1.96). These fragments have not been included in chimeric protein sequences because they could reduce the specificity in a serodiagnostic assay based on these new antigens.^31,32^ We also found that the antibodies from negative sera that recognized some of these fragments in the polypeptide microarray test could also identify pathogens that could be the cause of nonspecific reactions (Tables S2 and S3).

Looking at the results of the epitope mapping, epitopes that showed a *Z*-score > 2 and a *Z*-ratio > 1.96 when compared with negative samples could be diagnostically valuable in preventing false positives, differentiating the detected immunoglobulin isotype, or even providing information on the existence of seroconversion. In particular, peptides 20, 25, 48, 82, 144–146, 151, 220, 266–268, 286, and 297 in BmpA and peptides 14, 38, 39, 42–44, 88, 93, 112, 130, 236, 238, 240, 241, 243, 282, and 284 in BBK32 (Table S1). The results obtained from the microarray could be compared only with two previous studies. In one of them, 2 regions of B-cell epitopes, recognized by IgG, in BBK32 were identified (aa 16 to 30 and 51 to 80) by probing overlapping peptide libraries of BBK32 with serum from patients with early Lyme disease.^33^ In our study, the amino acid region from 16 to 30 only shared the last 4 amino acids (EMKE) with peptide number 14, which was identified as reactive in the microarray assay (Table S1). While the region that includes aa 51–80 (corresponding to peptides no. 38–49), identified as immunogenic by the previous study, showed reactivity with IgM in positive and negative serum samples in this study. As the authors noted, the patient population used in this study was derived from a single area; therefore, it is unclear if differences in the results of both studies could be due to the use of sera from different geographical locations^34^ or because the previous study identified epitopes with the use of early Lyme disease serum samples. Moreover, the results shown in this study identified a greater number of reactive peptides for IgM than for IgG immunoglobulin in BBK32, making this protein more specific for the diagnosis of early borreliosis. In the study from Tokarz et al., a chip for the diagnosis of tick-borne diseases was used to identify *B. burgdorferi* infections among other tick-borne pathogens employing 170,000 peptides from 62 proteins. One peptide from BBK32 was more frequently reactive to IgG in samples from patients with acute neuroborreliosis (EMKEESPGLFDKGNSILE).^35^ This peptide was also significantly identified in our peptide microarray by IgG from positive serum samples (Table S1).

Ten immunodominant fragments (5 for BmpA and BBK32) containing reactive epitopes (*Z*-ratio > 1.96) were chosen for the construction of chimeric proteins, allowing to evaluate the application of the obtained results. These fragments were characterized by a relatively high degree of amino acid sequence conservation within the *B. burgdorferi* s.l., which may contribute to the correct diagnosis of Lyme disease, regardless of which genospecies caused the infection (Tables 1 and 2). In most cases, the degree of sequence identity was over 80%; this applies in particular to *B. burgdorferi* s.s., *B. afzelii,* and *B. garinii*, i.e., the most significant genospecies.^36−38^

The identified fragments were divided in terms of their reactivity with antibody isotypes and combined into chimeric proteins dedicated to the detection of IgM or IgG. In addition, nonreactive fragments were introduced into chimeric protein sequences to ensure a molecular weight of at least 20 kDa, which allows for efficient biotechnological production, whereas the introduction of a flexible -GGG- sequence between individual peptides was intended to preserve the linear conformation of immunodominant fragments.

IgG-ELISA-B/32G showed relatively high sensitivity and specificity for the detection of IgG, 71% and 95%, respectively, and the AUC was 0.88. The sensitivity of the IgM-ELISA-B/32-M was also 71%; however, the specificity and AUC fell to 89% and 0.78, respectively. Student’s *t*-test showed a statistically significant difference in the absorbance obtained for both groups of sera. The lower reactivity of B/32-M is not surprising since IgM is the first-line antibody and has a lower concentration in the blood at its peak compared to that of IgG. Moreover, high levels of IgM are maintained in the blood for a very short time. It has been shown that enzyme immunoassays for the detection of IgM more often lead to false positive results, which may be caused by their lower affinity.^39−42^ Detection of IgM to recognize the early stage of Lyme disease is highly problematic and needs improvement. A study by Webber et al. (2019) estimated that false positives in IgM WB account for up to 53% of results, which causes overdiagnosis of Lyme diseases. This leads to the implementation of strong antibiotic therapy and delays correct diagnosis. Additionally, it must be noted that it is possible that some negative samples might have been diagnosed using the new epitopes identified but not using the previous standard assays, contributing to the results that were not significant, as well as the lack of clinical information about the groups of patients or the genospecies to which were exposed that should be considered.

Other recombinant antigens used in Lyme disease serodiagnosis have been OspC, which showed a sensitivity of 64% and a specificity of 100% and was used for detection of IgMs in early Lyme disease.^43,44^ Other ELISAs have been developed with promising results with VlsE (63% sensitivity for EM cases and 92% for later stages) to confirm Lyme disease by detecting specific antibodies induced by multiple pathogenic Borrelia genospecies. However, the tests detect mainly IgG antibodies and lack sensitivity during the early phase of Lyme disease.^45^ As we mentioned above, these recombinant proteins were added together in commercial WB or ELISA tests. Hence, in the present study, it is proposed that the development of chimeric proteins using the identified peptides may provide a diagnostic targeted to a single immunoglobulin isotype while avoiding cross-reactions and reducing the cost of producing serodiagnostic tests containing multiple recombinant proteins.

The difference in reactivity of *B. burgdorferi* s.l. antigens depending on the genospecies from which they were obtained is very common, even in proteins frequently used in commercial assays, such as DbpA and OspC, which are characterized by very high immunogenicity and diversity.^20,46^ Therefore, in Europe it is advisable to use several variants of recombinant antigens in diagnostic tests to accurately detect cases of Lyme disease caused by different genospecies. Although the literature reports suggest that *B. afzelii* and *B. garinii* are the dominant species in Europe, the results of research conducted in Poland do not provide a clear answer. Depending on the study area, the dominant species was *B. afzelii* or *B. burgdorferi* s.s.^36,47−50^ However, the latest and most extensive research in Poland by Strzelczyk et al. (2015) suggests that *B. burgdorferi* s.s. is the most widespread genospecies in Poland. Therefore, in this study epitope mapping was performed in BBK32 and BmpA from *B. burgdorferi* s.s.^51^

Reactivity of the BBK32 antigen obtained from the three most widespread genospecies of *B. burgdorferi* s.l. in Europe has been extensively studied. The sensitivity of IgG-ELISA-BBK32 ranges from 96% for BBK32 from *B. afzelii* to 21% for BBK32 from *B. burgdorferi* s.s. in late-stage Lyme disease patients and from 74% and 22%, respectively, when testing human sera for early Lyme disease. The specificity of these tests was high, 90–100% and 81–92% for late and early Lyme disease, respectively. Whereas in IgM-ELISA with EM patient samples, 4 to 13% of acute phase or convalescent-phase samples were positive, depending on the BBK32 variant used.^20,21^

WB revealed that despite the high degree of conservation of the BmpA protein sequence, its reactivity is also dependent on the genospecies it comes from. For WB assay based on BmpA from *B. afzelii* and *B. garinii,* the sensitivity values were 36.0 and 34.9%, respectively, and dropped to 13.9% for *B. burgdorferi* s.s. derived BmpA (BmpA_BB_). The specificity of this assay was 100%.^23^ IgG-ELISA-BmpA_BB_ showed a sensitivity of 45% and a specificity of 92%, while IgM-WB-BmpA had a very low sensitivity, which did not exceed 10%. In contrast, IgM-ELISA-BmpA_BB_ had a sensitivity of 43%.^23,52^

It seems that the selection of IgM- and IgG-specific epitopes has allowed the production of chimeric proteins that exhibit increased reactivity with a specific antibody isotype. This particularly applies to B/32-G, which is not reactive with anti-*B. burgdorferi* s.l. IgM. Student’s *t*-test did not show a statistically significant difference in the absorbance obtained for negative and positive sera, and the ROC analysis showed that the AUC is 0.646, which means that the adopted model is only slightly better than the random differentiation of both groups of sera (AUC = 0.5) (Table 3). This indicates that there is a very low number of epitopes specifically recognized by IgM antibodies in sequence B/32-G. The results obtained for B/32-M in IgG detection are not so clear. Student’s *t*-test showed a statistically significant difference in the absorbance values obtained for both groups of tested sera. In addition, the AUC calculated from the ROC is much higher at 0.722, not significantly different from that obtained for IgM-ELISA-B/32-M. The results suggest a lower reactivity of B/32-M with specific IgG in comparison to B/32-G. This is indicated not only by the lower AUC value but also by the median and average absorbance values. The same pool of positive sera was used in both tests, and the values obtained for B/32-M IgG detection are more than two times higher than those for B/32-G (Table 3). This suggests that the number of epitopes recognized by IgG antibodies in the B/32-M protein is reduced. The probable reason for a better result for B/32-M in the detection of nondedicated antibodies is the fact that IgG are present in the bloodstream in a much higher titer and show higher affinity. In addition, IgG targets a broader spectrum of epitopes, while IgM recognizes only those antigens and their fragments exposed in the early stages of infection.^39−42^

Although in this study we have analyzed only the design of a chimeric protein with the selected peptide regions (Figure 3), it is possible to use the peptides identified for the design and production of new chimeric proteins useful in serodiagnosis and to test their effectiveness using sera from different geographic origins and at different stages of the disease.

The polypeptide microarrays conducted here only allowed mapping linear epitopes, but other possible future approaches could study whether the conformation of these short peptide sequences is important in antibody binding. In addition, it was decided to construct multivalent chimeric proteins composed of these short identified immunodominant fragments instead of testing their reactivity as separate constructs because, usually, such short peptides do not have satisfactory diagnostic sensitivity^53^ and their isolation and purification can be very challenging even for moderately long peptides.^54^ Of course, the exception that immediately comes to mind is the C6 peptide, where tests based on it are characterized by even 100% sensitivity in late Lyme disease. However, Arnaboldi et al.’s (2022) studies suggest that it is very difficult to label such a peptide, even if it shows high reactivity during mapping. Another research group mapped the linear epitopes of ErpP, BBH32, and FlaB antigens and then checked the reactivity of the identified immunodominant fragments by ELISA. The FlaB fragment provided the highest sensitivity; tests based on it reached a sensitivity of 37.6%, which is insufficient for the effective diagnosis of Lyme disease. Only the addition of a fragment derived from VlsE increased the sensitivity of ELISA.^55^

In this study, the tertiary structure of B/32-M and B/32-G recombinant chimeric proteins may not contain any naturally occurring structural epitopes, as noncontiguous protein sequences in the native proteins have been joined together into a single amino acid chain. In addition, sequences -GGG- were introduced, forcing a linear conformation for the exposure of the epitopes to the antibodies, maintaining the form in which they were recognized on the peptide microarray. Therefore, it is possible that proteins of this type will also be useful in WB tests, where antigens are used in a denatured form. In ELISA, it may be advisable to use chimeric proteins composed of larger fragments of antigens, so as to at least partially preserve their conformational epitopes.^56,57^

## Conclusions

The results presented indicate that the epitopes identified could be useful for the design of chimeric proteins that may contribute to the development of new diagnostic tools that improve the effectiveness of Lyme disease diagnosis. In addition, B/32-G and B/32-M are the first attempts to design chimeric proteins from BBK32 and BmpA epitope mapping data. This study highlighted that the appropriate selection of linear epitopes allows the construction of chimeric proteins showing increased reactivity with a specific antibody isotope, which can improve the diagnosis of early Lyme disease, avoiding, at the same time, possible nonspecific cross reactions.

## Material and Methods

### Human Serum Samples

In this study 384 human sera (148 IgG positive, 48 IgM positive, 8 IgM/IgG positive, 180 negative) were used. All were obtained from the National Institute of Public Health NIH - National Research Institute (Warsaw, Poland) during routine borreliosis screening, in accordance with the ethical standards of this Institute and with the World Medical Association Declaration of Helsinki. Anonymized information about each sample included only the date of collection and the titer of anti-*B. burgdorferi* s.l. antibodies. IgG and IgM levels were redetermined using a commercial ELISA (Borrelia plus VlsE, Euroimmun and Borrelia Select: recombinant antigens with OspC, Euroimmun, Lübeck Germany). The presence of specific anti-*B. burgdorferi* s.l. IgG and IgM antibodies were further confirmed using a commercial WB (EUROLINE Borrelia, Euroimmun, Lübeck, Germany).

### Antibody IgG and IgM Binding to BBK32 and BmpA Epitopes Microarray

The *B. burgdorferi* s.s. B31 BBK32 (GenBank ID: AAC66134.1) and BmpA (GenBank ID: AAC66757.1) peptide microarrays elongated with neutral GSGSGSG linkers at the C- and N-terminus and translated, respectively, into 334 and 322 different overlapping 15 amino acid (aa) peptides (peptide–peptide overlap of 14 aa) were printed (668 and 644 peptide spots for BBK32 and BmpA array copies) at PEPperCHIP Immunoassay, PEPperPRINT, Germany. Serum samples positive for IgG (*N* = 22), positive for IgM (*N* = 14), positive for both (IgG+/IgM+) (*N* = 8), and negative (*N* = 30) were used to identify immunoreactive regions in *B. burgdorferi* s.s. BBK32 and BmpA proteins. Subsequently, high-resolution epitope mapping of each protein was performed. The peptide microarray was assembled in an incubation tray and blocked with 1% (w/v) bovine serum albumin (BSA) in PBS pH 7.4 with 0.05% (v/v) Tween-20 (PBST) for 30 min at room temperature (RT). After it was washed with PBST three times, the array was incubated with pooled sera diluted 1:100 in blocking solution overnight at 4 °C. The next day, each microarray slide was washed two times and incubated with goat antihuman IgM (μ chain) Alexa Fluor 647 (Invitrogen, Paisley, UK) diluted 1:2000 and goat antihuman IgG Fc Cross-Adsorbed Alexa DyLight 550 (Invitrogen, Paisley, UK) diluted 1:1500 in blocking solution for 45 min at RT. The array was washed again four times, dissembled from the tray, dipped in dipping buffer (Tris 1 mM, pH 7.4), and dried with centrifugation for 1 min at 190*g*. The resulting array was scanned with a GenePix personal 4100a microarray scanner (Molecular Devices, San José, CA, USA), and GenePix Array List (GAL) files supplied by the microarray slide manufacturer were used for image analysis. The median fluorescent signal intensity of each spot was extracted using MAPIX software (Molecular Devices, San José, CA, USA).

### Data Analysis and Peptide Characterization

For data analysis, the intensity of raw fluorescence signal in each spot corresponded to the median signal intensity, and it was subtracted from the median background intensity, then averaged across duplicate spots.^58^ The resulting signals were normalized with a *Z*-Score.^59,60^*Z*-Score = (intensityP – mean intensityP1...P*n*)/SD P1...P*n*, where P is any BBK32 or BmpA peptide on the microarray, and P1...P*n* represent the aggregate measure of all peptides. Heatmaps of IgG, IgM, and mixed antibodies bound to the peptides were visualized using a *Z*-Score heatmap (http://www.heatmapper.ca/expression/), where peptides that showed *Z*-scores > 2 were considered significantly reactive. *Z*-ratios from each immunoglobulin isotype were used for comparisons between peptides from positive and negative serum groups and were calculated by taking the difference between the averages of the observed peptide *Z*-scores and dividing them by the SD of all peptide *Z*-score differences. A *Z*-ratio of ±1.96 was inferred as significant (*P* < 0.05). Analysis was focused on epitopes with a *Z*-ratio > 1.96 when comparing the peptide reactivity in the positive serum sample group to the same peptide in the negative serum sample group (all data are available in Table S2 in the Supporting Information).

### Analysis of the Amino Acid Sequence of Reactive Fragments

ClustalX2 software was used to assess the degree of conservation of amino acid sequences of the selected immunodominant fragments. Amino acid sequences from five *B. burgdorferi* s.l. genospecies were compared, i.e., *B. burgdorferi* s.s. (4 strains: *B. burgdorferi* s.s.: B31; JD1; ZS7; N40); *B. afzelii* (4 strains: Pko; K78; HLJ01; Tom3107); *B. garinii* (4 strains: 20047; NMJW1; BgVir; PBr); *B. bavariensis* (1 strain: PBi), and *B. spielmanii* (1 strain: A14S). In order to identify microorganisms that could be the source of nonspecific interactions between individual peptides and antibodies contained in negative sera, the Protein Basic Local Alignment Search Tool^61^ was used to search for sequences identical in length of at least 6 amino acids^62^ with cross-reactive fragments contained in the tested proteins.

### Construction of Recombinant Plasmids

Once the peptides or reactive regions recognized by each immunoglobulin isotype were identified, two chimeric proteins were designed and produced. Genes encoding the BmpA-BBK32-G (B/32-G) and BmpA-BBK32-M (B/32-M) chimeric proteins dedicated to detection of IgG and IgM, respectively, have been synthesized by GeneScript (Rijswijk, Netherlands) and cloned into a pUET1 plasmid using *Bam*HI and *Hin*dIII restriction sites.^63^ In this way, recombinant plasmids encoding chimeric proteins containing a His_6_-tag at both ends, allowing for their purification by metal affinity chromatography, were obtained. Nucleotide sequences of the recombinant plasmids were confirmed by DNA sequencing (Genomed, Poland).

### Expression and Purification of Chimeric Proteins

B/32-G and B/32-M were produced in *E. coli* BL21(DE3) pLysS cells. *E. coli* cells transformed with the appropriate recombinant plasmid were grown in LB medium supplemented with 100 μg/mL ampicillin and 34 μg/mL chloramphenicol, until the optical density at λ = 600 nm reached 0.4. Protein production was then induced with isopropyl-β-d-thiogalactopyranoside (IPTG) at a final concentration of 1 mM, and cells were incubated with vigorous shaking at 37 °C for 18 h. After this time, cells were harvested by centrifugation. The pellet was resuspended in binding buffer [20 mM Tris; 500 mM NaCl; 1 mM PMSF, 0.1% (v/v) Triton X-100 (pH 7.9–9.7)], and cells were lysed by sonication. Proteins were purified in a one-step metal affinity chromatography using His·Bind Resin (Novagen, Madison, WI, USA) under native conditions following the manufacturer’s instructions. Chimeric proteins were eluted from the resin by increasing the imidazole concentration. After elution, proteins were dialyzed into storage buffer (50 mM Tris–HCl; 150 mM). The purity of the obtained chimeric protein preparations was verified by 12% SDS-PAGE, and the results were quantified by Image Lab software (Bio-Rad, Hercules, CA, USA). Protein concentration was measured with a Bradford Assay Kit according to the manufacturer’s instructions (Bio-Rad, Hercules, CA, USA) using BSA as a standard.

### ELISA

ELISA was used to assess the reactivity of anti-*B. burgdorferi* s.l. antibodies contained in human sera with constructed chimeric proteins. MaxiSorp microtiter plates (Nunc, Waltham, MA, USA) were coated with antigens at 10 μg/mL in a coating buffer (200 mM carbonate buffer, pH 9.6). After overnight incubation at 4 °C, the plates were washed four times with washing buffer (50 mM Tris; 150 mM NaCl; 0.1% (v/v) Tween-20, pH = 7.4). Then plates were blocked for 1 h at 37 °C in blocking buffer [3% (w/v) nonfat milk, 0.1% (v/v) Tween-20 in PBS]. The wells were washed again and incubated for 1 h at 37 °C with human serum diluted 1:100 in blocking buffer. After washing, peroxidase-labeled antibodies directed against human IgG or IgM (Jackson ImmunoResearch, #109-035-003 and #109-035-129 Ely, UK) were added, diluted 1:32,000 and 1:16,000, respectively. 3,3′,5,5′-tetramethylbenzidine (TMB, Thermo Scientific, Waltham, MA, USA) was used as a substrate for the detection of the formed immune complexes; absorbances were measured at λ = 450 nm.

### Statistical Analysis

For all data manipulation, GraphPad Prism software was used (GraphPad Prism, Version 9, San Diego, CA, USA). The presence of a statistically significant difference in the absorbance obtained for the negative and positive sera was determined with the use of a Student’s *t*-test. Statistical significance was considered when the p value was below 0.05. Receiver operating characteristic (ROC) was performed to obtain area under the curve (AUC), optimal cutoff, and the sensitivity and the specificity of the assays based on chimeric proteins. The optimal cutoff was determined as the point on the ROC curve closest to (0,1) corner.^64^

## Supporting Information Available

BmpA and BBK32 overlapping peptides identified as reactive in each of the study group; production of chimeric proteins; Western blot using anti-His-tag antibodies; and potential sources of nonspecific reactions (PDF)
*Z*-score and *Z*-ratio for particular peptides (XLSX)

## Abbreviations Used

EM: erythema migrans
s.s.: sensu stricto
s.l.: sensu lato
WB: Western blot
WCL: whole-cell lysate
