# Supplementary material for: Epitope Mapping of BmpA and BBK32 Borrelia burgdorferi Sensu Stricto Antigens for the Design of Chimeric Proteins with Potential Diagnostic Value
Source: ACS Infect Dis. 2023 Oct 7;9(11):2160–72. doi: 10.1021/acsinfecdis.3c00258 (PMC10722512; doi:10.1021/acsinfecdis.3c00258)
Supplement: Supplementary file 1 — id3c00258_si_001.pdf [file id3c00258_si_001.pdf]

Supplementary Information: Epitope mapping of BmpA and BBK32 *B. burgdorferi* sensu stricto antigens for the design of chimeric proteins with potential diagnostic value

Weronika Grąźlewska[1,2], Lucyna Holec-Gąsior[1], Karolina Sołowińska[1], Tomasz Chmielewski[3], Beata Fiecek[3], Marinela Contreras[2*]

[1]Department of Molecular Biotechnology and Microbiology, Faculty of Chemistry, Gdańsk University of Technology, 80-233 Gdańsk, Poland;

[2] SaBio, Instituto de Investigación en Recursos Cinegéticos IREC-CSIC-UCLM-JCCM, 13005, Ciudad Real, Spain,

[3] Department of Parasitology and Diseases Transmitted by Vectors, National Institute of Public Health NIH - National Research Institute, 00-791 Warsaw, Poland

* Corresponding author: marinela.contreras@csic.es; marinelacr@hotmail.com

Table S1. BmpA and BBK32 overlapping peptides identified as reactive in each of the study groups.

BmpA			Serum groups					
**Peptide nº**	**Position in protein sequence**	**Peptide sequence**	**IgG Negative (Control)**	**IgG Positive**	**IgM positive**	**IgM negative (Control)**	**IgG mix (IgG+/IgM+)**	**IgM mix (IgG+/IgM+)**
17	27-41	EIPKVSLIIDGTFDD						X
20	30-44	KVSLIIDGTFDDKSF					X*ϕ	
25	35-49	IDGTFDDKSFNESAL						X*ϕ
26	36-50	DGTFDDKSFNESALN			X			X
40	50-64	NGVKKVKEEFKIELV	X					
47	57-71	EEFKIELVLKESSSN	X	X*			X	
48	58-72	EFKIELVLKESSSNS		X*				
54	64-78	VLKESSSNSYLSDLE	X					
59	69-83	SSNSYLSDLEGLKDA						*
60	70-84	SNSYLSDLEGLKDAG						*
61	71-85	NSYLSDLEGLKDAGS	X					
62	72-86	SYLSDLEGLKDAGSD						X*
64	74-88	LSDLEGLKDAGSDLI	X					X*
82	92-106	GYRFSDVAKVAALQN			X*			
93	103-117	ALQNPDMKYAIIDPI				X		
94	104-118	LQNPDMKYAIIDPIY				X		
95	105-119	QNPDMKYAIIDPIYS				X		
96	106-120	NPDMKYAIIDPIYSN				X		
97	107-121	PDMKYAIIDPIYSND			X	X		X
98	108-122	DMKYAIIDPIYSNDP				X		
99	109-123	MKYAIIDPIYSNDPI				X		

BmpA			Serum groups					
**Peptide nº**	**Position in protein sequence**	**Peptide sequence**	**IgG Negative (Control)**	**IgG Positive**	**IgM positive**	**IgM negative (Control)**	**IgG mix (IgG+/IgM+)**	**IgM mix (IgG+/IgM+)**
100	110-124	KYAIIDPIYSNDPIP				X		
102	112-126	AIIDPIYSNDPIPAN			X	X		X
103	113-127	IIDPIYSNDPIPANL			X	X		
106	116-130	PIYSNDPIPANLVGM				X		
109	119-133	SNDPIPANLVGMTFR	X					
110	120-134	NDPIPANLVGMTFRA	X					
112	122-136	PIPANLVGMTFRAQE	X					
116	126-140	NLVGMTFRAQEGAFL	X					
142	152-166	GKIGFLGGIEGEIVD				X	X	
144	154-168	IGFLGGIEGEIVDAF					X*	
145	155-169	GFLGGIEGEIVDAFR					X*	
146	156-170	FLGGIEGEIVDAFRY					X*ϕ	
149	159-173	GIEGEIVDAFRYGYE						X
151	161-175	EGEIVDAFRYGYEAG		X*				
173	183-197	IKISTQYIGSFADLE						X*
186	196-210	LEAGRSVATRMYSDE					X	
220	230-244	KELGSGHYIIGVDED						X*
221	231-245	ELGSGHYIIGVDEDQ			X			X
228	238-252	IIGVDEDQAYLAPDN				X		
242	252-266	NVITSTTKDVGRALN	X					
255	265-279	LNIFTSNHLKTNTFE	X					
257	267-281	IFTSNHLKTNTFEGG	X					

BmpA			Serum groups					
Peptide nº	Position in protein sequence	Peptide sequence	IgG Negative (Control)	IgG Positive	IgM positive	IgM negative (Control)	IgG mix (IgG+/IgM+)	IgM mix (IgG+/IgM+)
261	271-285	NHLKTNTFEGGKLIN	X					
265	275-289	TNTFEGGKLINYGLK						*ϕ
266	276-290	NTFEGGKLINYGLKE						X*ϕ
267	277-291	TFEGGKLINYGLKEG						X*ϕ
268	278-292	FEGGKLINYGLKEGV						X*ϕ
269	279-293	EGGKLINYGLKEGVV		*				
286	296-310	VRNPKMISFELEKEI					X*ϕ	
297	307-321	EKEIDNLSSKIINKE		X*				
299	309-323	EIDNLSSKIINKEII				X		
302	312-326	NLSSKIINKEIIVPS	X					
304	314-328	SSKIINKEIIVPSNK	X					
309	319-333	NKEIIVPSNKESYEK	X					
317	327-339	NKESYEKFLKEFIGS	X					
BBK32			Serum groups					
Peptide nº	Position in protein sequence	Peptide sequence	IgG Negative (Control)	IgG Positive	IgM positive	IgM negative (Control)	IgG mix (IgG+/IgM+)	IgM mix (IgG+/IgM+)
14	27-41	EMKEESPGLFDKGNS		X*				
23	36-50	FDKGNSILETSEESI	X					
27	40-54	NSILETSEESIKKPM	X					
35	48-62	ESIKKPMNKKGKGKI			*			
38	51-65	KKPMNKKGKGKIARK			X*			
39	52-66	KPMNKKGKGKIARKK			X*			
40	53-67	PMNKKGKGKIARKKG				X		

BBK32			Serum groups					
Peptide nº	Position in protein sequence	Peptide sequence	IgG Negative (Control)	IgG Positive	IgM positive	IgM negative (Control)	IgG mix (IgG+/IgM+)	IgM mix (IgG+/IgM+)
42	55-69	NKKGKGKIARKKGKS			X*			
43	56-70	KKGKGKIARKKGKSK			X*			
44	57-71	KGKGKIARKKGKSKV			X*			
45	58-72	GKGKIARKKGKSKVS			X	X		
46	59-73	KGKIARKKGKSKVSR				X		
47	60-74	GKIARKKGKSKVSRK				X		
49	62-76	IARKKGKSKVSRKEP				X		
86	99-113	EEESLKTELLKEQSE						ϕ
88	101-115	ESLKTELLKEQSETR		X*				
93	106-120	ELLKEQSETRKEKIQ		X*				
100	113-127	ETRKEKIQKQQDEYK		*				
112	125-139	EYKGMTQGSLNSLSG		X*				
122	136-149	NSLSGESGELEEPIE	X					
125	139-152	SGESGELEEPIESNE						X
127	141-154	ESGELEEPIESNEID	X	X*				X
130	143-157	ELEEPIESNEIDLTI		X*			*	X*ϕ
134	147-161	PIESNEIDLTIDSDL						*
135	148-162	IESNEIDLTIDSDLR	X					
136	149-163	ESNEIDLTIDSDLRP		X*		X		
157	170-184	IAGSNSISYTDEIEE					X	
160	173-187	SNSISYTDEIEEEDY	X				X	
161	174-188	NSISYTDEIEEEDYD	X					

BBK32			Serum groups					
**Peptide nº**	**Position in protein sequence**	**Peptide sequence**	**IgG Negative (Control)**	**IgG Positive**	**IgM positive**	**IgM negative (Control)**	**IgG mix (IgG+/IgM+)**	**IgM mix (IgG+/IgM+)**
162	175-189	SISYTDEIEEEDYDQ				X		
163	176-190	ISYTDEIEEEDYDQY				X		
164	177-191	SYTDEIEEEDYDQYY	X		X	X		
165	178-192	YTDEIEEEDYDQYYL				X		
166	179-193	TDEIEEEDYDQYYLD	X	X		X	X∗ϕ	
167	180-194	DEIEEEDYDQYYLDE			X	X	X	
168	181-195	EIEEEDYDQYYLDEY	X	X	X∗		X	
169	182-196	IEEEDYDQYYLDEYD	X	X	X∗	X	X	
170	183-197	EEEDYDQYYLDEYDE	X	X	X	X	X	
171	184-198	EEDYDQYYLDEYDEE	X	X	X	X	X	
172	185-199	EDYDQYYLDEYDEED	X	X	X	X		
173	186-200	DYDQYYLDEYDEEDE	X		X	X		X
174	187-201	YDQYYLDEYDEEDEE	X	X	X	X		
175	188-202	DQYYLDEYDEEDEEE	X		X	X	X	X
176	189-203	QYYLDEYDEEDEEEI	X		X	X		X
177	190-204	YYLDEYDEEDEEEIR	X	X	X	X	X	
178	191-205	YLDEYDEEDEEEIRL	X		X	X	X	X
179	192-206	LDEYDEEDEEEIRLS	X			X		
180	193-207	DEYDEEDEEEIRLSN			X	X		X
181	194-208	EYDEEDEEEIRLSNR		X∗			X	X
184	197-211	EEDEEEIRLSNRYQS		X				X
185	198-212	EDEEEIRLSNRYQSY		X				X∗ϕ

BBK32			Serum groups					
Peptide nº	Position in protein sequence	Peptide sequence	IgG Negative (Control)	IgG Positive	IgM positive	IgM negative (Control)	IgG mix (IgG+/IgM+)	IgM mix (IgG+/IgM+)
187	200-214	EEEIRLSNRYQSYLE						X
236	249-263	LDNFAKAKAKEEAAK			*			X*ϕ
238	251-265	NFAKAKAKEEAAKFT			*			X*ϕ
239	252-266	FAKAKAKEEAAKFTK			*			*
240	253-267	AKAKAKEEAAKFTKE			*			X*ϕ
241	254-268	KAKAKEEAAKFTKED			*			X*ϕ
243	256-270	KAKEEAAKFTKEDLE			*			X*
279	292-306	YINDTHAKRKLENIE						*
282	295-309	DTHAKRKLENIEAEI						X*ϕ
284	297-311	HAKRKLENIEAEIKT						X*ϕ

x, reactive peptides with Z-score >2. *, peptides that showed significant differences with Z-ratio >1.96 when are compared with the negative immunoglobulin isotype group (Control), ϕ peptides that showed significant differences with Z-ratio >1.96 when are compared with positive IgG or IgM samples depending on the immunoglobulin isotype.
Serum groups= IgG in the Negative samples (Control); IgG in the samples positive to IgG (IgG Positive); IgM in the samples positive to IgM (IgM Positive); IgM in the Negative samples (Control); IgG in samples positive to IgG and IgM (IgG mix (IgG+/IgM+)); IgM in samples positive to IgG and IgM (IgM mix (IgG+/IgM+)).

Table S2 Z-score and Z-ratio for particular peptides (excel file)

Table S3 Potential sources of non-specific reactions occurring during epitope mapping of the BmpA antigen of *B. burgdorferi* s.s. B31

IF No	**Fragment sequence**	**Cross-reactive Ab isotype**	**Length [aa]**	**Potential source of cross-reactions**
1	^{71}NSYLSDLEGLKDAGS^{85}	IgG	15	• RFB (*B. recurrentis, B. hispanica*)<br>• *T. pallidum*
2	^{107}PDMKYAIIDPIYSNDPIP ANLVGMTFRA^{134}	IgM/IgG	28	• RFB (*B. hermsii; B. hisanica*)
3	^{180}NKDIKISTQYIGSF^{193}			• RFB (*B. hermsii, B. miyamotoi*)
4	^{225}AIEVAKELGSGHYII^{239}	IgM	15	• RFB (*B. hermsii, B. miyamotoi*)<br>• *E. coli*<br>• *Streptococcus lactarius; Streptococus parasuis; Streptococus suis*<br>• *T. pallidum*
5	^{252}NVITSTTKDVGRALNIF TSNHLKTNTFEGGKLINY GLKEG^{291}	IgG	40	• RFB (*B. miyamotoi*)<br>• *E. coli*<br>• *Listeria monocytogenes*
6	^{312}NLSSKIINKEIIVPSNKES YEK^{333}	IgG	22	• RFB (*B. hisanica; B. duttonii; B. recurrentis; B. hermsii*)<br>• *E. coli*<br>• *T. pallidum*

Table S4 Potential sources of non-specific reactions occurring during epitope mapping of the BBK332 antigen of *B. burgdorferi* s.s. B31

IF No	Fragment sequence	Cross-reactive Ab isotype	Length [aa]	Potential source of cross-reactions
1	^{36}FDKGNSILETSEESIKKPM^{54}	IgG	19	• *Treponema* sp<br>• *E. coli*<br>• *Lachnospiraceae bacterium*
2	^{95}VILEEESLKTELLK^{109}	IgM	15	• RFB (*B. miyamotoi; B. parkeri; B. hermsii*)<br>• *E. coli*<br>• *Clostridioides difficile*
3	^{135}NSLSGE^{140}	IgG	6	• *E. coli*<br>• Inflenza A virus<br>• *Salmonella enterica*
4	^{147}PIESNEIDLTIDSDLR^{162}	IgM/IgG	16	• RFB (*B. miyamotoi; B. recurrentis; B. hispanica*)<br>• *E. coli*<br>• *Lachnospiraceae bacterium*<br>• *Corynebacterium ulcerans*
5	^{174}NSISYTDEIEEEDYDQYYLDEYDEEDEEEIRL^{205}	IgG	32	• RFB (*B. miyamotoi; B. recurrentis; B. hispanica*)<br>• *E. coli*<br>• Influenza B virus<br>• *Plasmodium vivax*<br>• *Dibothriocephalus latus*<br>• *Prevotella copri*<br>• *Bartonella koehlerae*<br>• *Moraxella lacunata*

				• *Clostridium celatum; Clostridium neonatale; Clostridium disporicum; Clostridium intestinale*

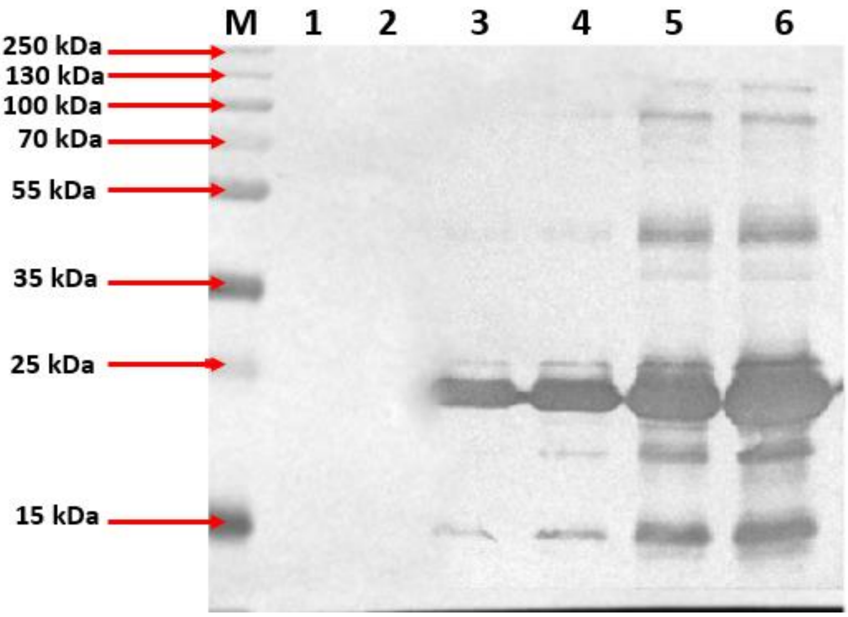

Figure S1 Production of BmpA-BBK32-G. Western blot using anti-His-tag antibodies.

Lane M - Protein ladder PageRuler™ Plus Prestained Protein Ladder (Thermo Scientific #26619)

Lane 1 – Negative control, *E. coli* BL21(DE3)pLacI + pUET1

Lane 2 –*E. coli* BL21(DE3)pLacI + pUET1-BmpA-BBK32-G, before induction

Lane 3 - *E. coli* BL21(DE3)pLacI + pUET1-BmpA-BBK32-G, 2h after induction

Lane 4 - *E. coli* BL21(DE3)pLacI + pUET1-BmpA-BBK32-G, 4h after induction

Lane 5 - *E. coli* BL21(DE3)pLacI + pUET1-BmpA-BBK32-G, 6h after induction

Lane 6 - *E. coli* BL21(DE3)pLacI + pUET1-BmpA-BBK32-G, 18h after induction

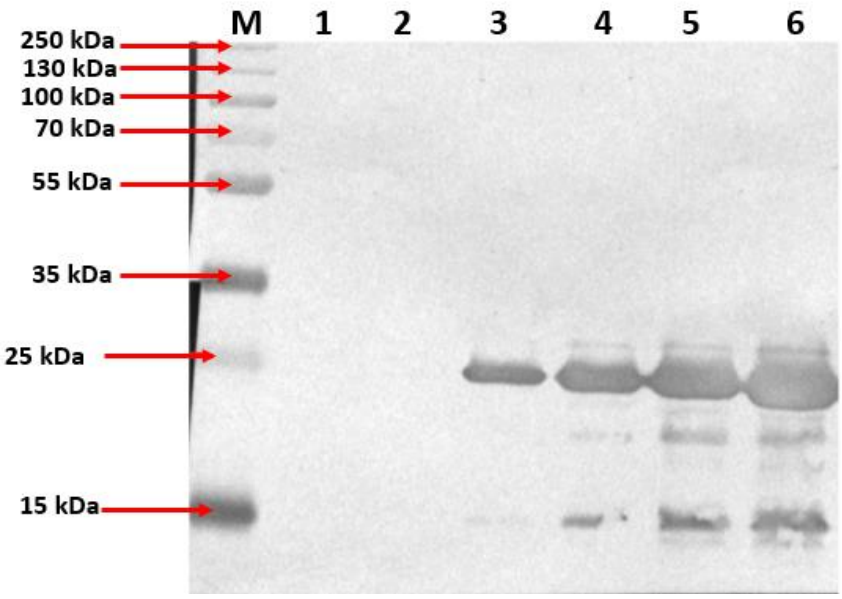

Figure S2 Production of BmpA-BBK32-M. Western blot using anti-His-tag antibodies.

Lane M - Protein ladder PageRuler™ Plus Prestained Protein Ladder (Thermo Scientific #26619)

Lane 1 – Negative control, *E. coli* BL21(DE3)pLacI +pUET1

Lane 2 –*E. coli* BL21(DE3)pLacI + pUET1-BmpA-BBK32-M, before induction

Lane 3 - *E. coli* BL21(DE3)pLacI + pUET1-BmpA-BBK32-M, 2h after induction

Lane 4 - *E. coli* BL21(DE3)pLacI + pUET1-BmpA-BBK32-M, 4h after induction

Lane 5 - *E. coli* BL21(DE3)pLacI + pUET1-BmpA-BBK32-M, 6h after induction

Lane 6 - *E. coli* BL21(DE3)pLacI + pUET1-BmpA-BBK32-M, 18h after induction
